# Electric-field-based dosing for TMS

**DOI:** 10.1162/imag_a_00106

**Published:** 2024-03-11

**Authors:** Ole Numssen, Philipp Kuhnke, Konstantin Weise, Gesa Hartwigsen

**Affiliations:** Lise Meitner Research Group Cognition and Plasticity, Max Planck Institute for Human Cognitive and Brain Sciences, Leipzig, Germany; Methods and Development Group Brain Networks, Max Planck Institute for Human Cognitive and Brain Sciences, Leipzig, Germany; Wilhelm Wundt Institute for Psychology, Leipzig University, Leipzig, Germany; Department of Clinical Medicine, Aarhus University, Aarhus, Denmark

**Keywords:** cognition, cortical stimulation threshold, dosing, NIBS, TMS, tES, variability

## Abstract

Transcranial magnetic stimulation (TMS) is an invaluable non-invasive brain stimulation (NIBS) technique to modulate cortical activity and behavior, but high within- and between-participant variability limits its efficacy and reliability. Here, we explore the potential of electric field (e-field) based TMS dosing to reduce its variability and discuss current challenges as well as future pathways. In contrast to previous dosing approaches, e-field dosing better matches the stimulation strength across cortical areas, both within and across individuals. Challenges include methodological uncertainties of the e-field simulation, target definitions, and comparability of different stimulation thresholds across cortical areas and NIBS protocols. Despite these challenges, e-field dosing promises to substantially improve NIBS applications in neuroscientific research and personalized medicine.

## 1 Introduction

Non-invasive brain stimulation (NIBS) techniques, such as transcranial magnetic stimulation (TMS;Pascual-Leone et al., 2000), have emerged as invaluable tools for modulating brain activity in both healthy individuals (Walsh & Cowey, 2000) and psychiatric patients (Burt et al., 2002). Notably, TMS has received FDA approval as a therapeutic intervention for several disorders (e.g.,O’Reardon et al., 2010). However, high variability of the stimulation effects within and across individuals limits strong conclusions about structure-function relationships (Caulfield & Brown, 2022;Numssen, van der Burght, et al., 2023). Likewise, the effect sizes in TMS studies are often small (Beynel et al., 2019). Consequently, there is an ongoing debate about the validity and reliability of different TMS protocols in research and treatment settings (e.g.,Hartwigsen et al., 2015;Sandrini et al., 2011). Recent studies emphasize that differences in the individual responsiveness to NIBS strongly affect the outcomes and thus explain large parts of the observed variance (e.g.,Hamada et al., 2013). One key factor determining NIBS effects, both for primary sensory-motor regions (Sasaki et al., 2018) and higher association areas (Lee et al., 2021), is the stimulation strength. Throughout this Perspective, we utilize the term*dosage*to refer to the strength of the TMS-induced cortical stimulation, targeted towards researchers and clinicians applying conventional TMS with available hard- and software. A broader view of potential influence factors is provided in the*Remaining challenges of e-field-based dosing*section. We also refer the reader toPeterchev et al. (2012)for an in-depth discussion of dosing parameters apart from the local stimulation strength. Currently, the gold standard for dosing across the brain is based on the individual motor cortex excitability and quantified via the motor threshold (MT) (Turi et al., 2021).

**Fig. 1.**
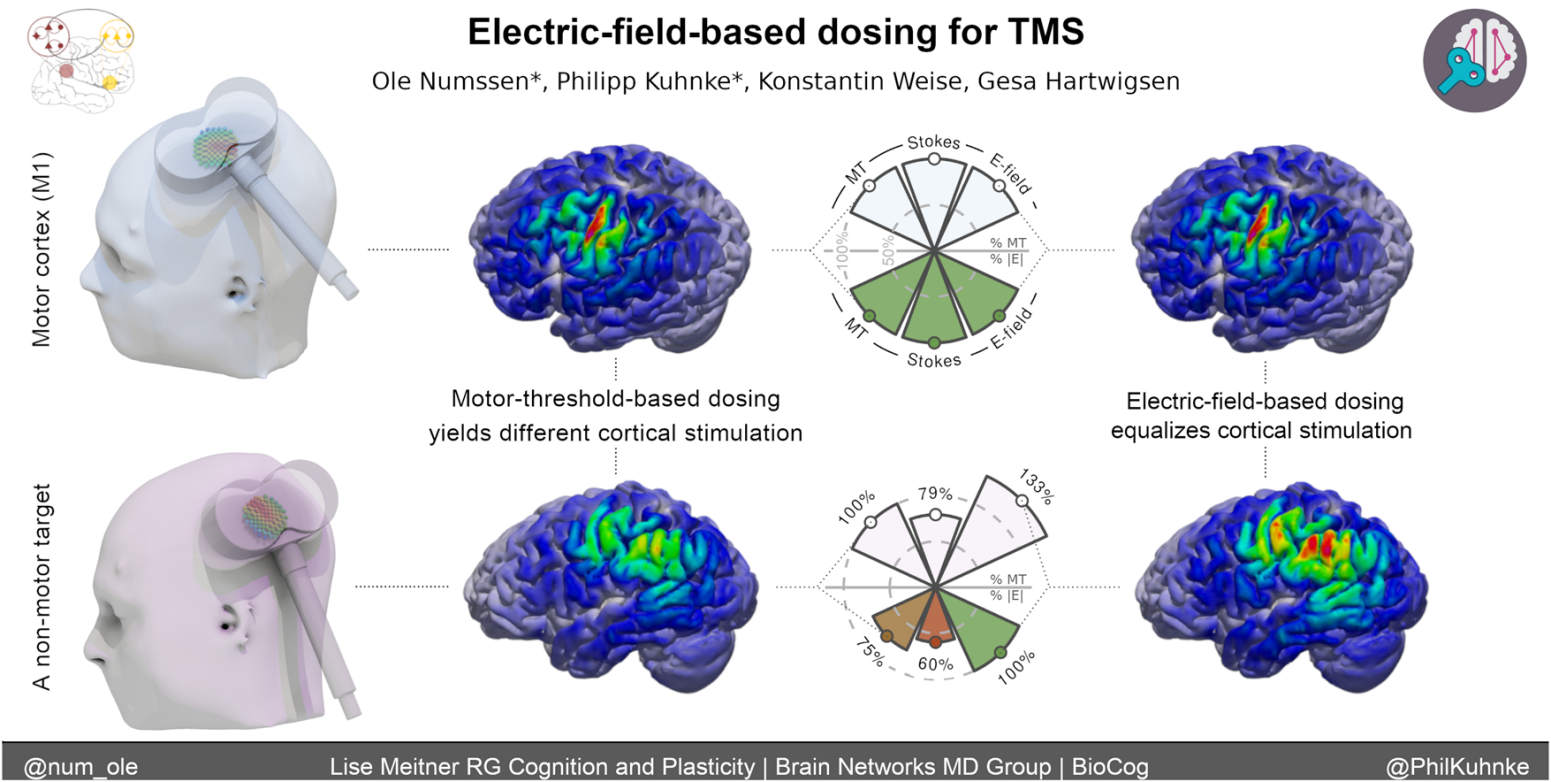
Motor threshold based dosing applies the same*stimulator intensity*across different cortical targets instead of calibrating the cortical*stimulation strength*. In contrast, electrical-field-based dosing equalizes the realized*stimulation strength*across brain regions.

Here, we identify shortcomings of the MT-based dosing approach across various stimulation targets and suggest an alternative strategy. To this end, we present experimental data directly comparing different dosing approaches in the same set of individuals. Critically, standard MT-based dosing strategies fail to consider the actual level of stimulation of the cortical target due to their focus on the*stimulator*intensity (e.g., 50% maximum stimulator output; MSO). In contrast, we highlight the potential of TMS dosing based on the*cortical stimulation*itself, quantified via the induced electric field (Fig. 1;Caulfield et al., 2021a;Dannhauer et al., 2022;Kuhnke, Numssen, et al., 2023;Weise, Numssen, et al., 2022). Throughout this Perspective,*stimulator intensity*refers to the intensity that is set at the stimulator device (e.g., 50% MSO). In contrast,*stimulation strength*refers to the cortical stimulation exposure, quantified by the e-field strength (e.g., 100 V/m). It is essential to note that the TMS-induced e-field shows limited focality, rendering the exclusive stimulation of a single cortical location, without any off-target stimulation, impossible (Kuhnke et al., 2020). In our prior discussions (Numssen, van der Burght, et al., 2023), we explored this limitation of TMS and other NIBS approaches and presented strategies to address these constraints. Here, we shift our focus to the singular cortical target—a spatially delimited region within the cortical gray matter. This specific region is the intended focal point for effective stimulation, guided by the presumed importance of this cortical structure in relation to a distinct functional domain. Consequently, we introduce the term “TMS-induced effect” to broadly characterize a cortical modulation that results in measurable effects on a designated outcome variable (seeHartwigsen & Silvanto (2022)for an in-depth discussion). This term encapsulates the observable impact stemming from the targeted cortical region, reflecting our focus on a localized, functionally relevant dosing approach.

Recent methodological advances have enabled the computational simulation of TMS-induced e-fields (e.g., SimNIBS,Puonti et al., 2020;Thielscher et al., 2015; ROAST,Huang et al., 2019), yielding the foundation for a biophysiologically informed dosing strategy. Calibrating the cortical stimulation exposure to the individual participant and brain region removes a critical variance source of TMS studies: the intra- and inter-individual variability in cortical stimulation exposure due to anatomical differences (Caulfield et al., 2021b;Kuhnke, Numssen, et al., 2023). This Perspective utilizes single- and group-level analyses of cortical stimulation from TMS to identify shortcomings of dosing approaches that do not take the cortical stimulation into account. We reason that our e-field-based dosing approach may also inform other transcranial electric and magnetic NIBS approaches that rely on induced electric fields (Peterchev et al., 2012), such as transcranial direct/alternating current stimulation (Jiang et al., 2020;Kasten et al., 2019;Laakso et al., 2019;Wischnewski et al., 2021) or temporal interference stimulation (Esmaeilpour et al., 2021). Although stimulation principles differ (e.g., supra- vs. sub-threshold stimulation), it was shown that differences in individual morphology affect the cortical stimulation exposure to a similar or even higher degree (Weise, Wartman, et al., 2022).

While e-field-based dosing represents an important step towards more reliable and predictable NIBS outcomes, several links between stimulation intensity and outcome variables remain under-researched, limiting the full potential of this approach. This includes rigorous and detailed assessments of behavioral and clinical relevance. We discuss these issues in detail, including the uncertainties associated with e-field computations and the challenges associated with transitioning from single-pulse to repetitive TMS thresholds. Addressing these knowledge gaps will help unlock the currently unexploited potential of TMS—and potentially other NIBS approaches—for the study of human cognition and the treatment of neurological and psychiatric disorders.Box 1provides an overview of common implicit assumptions when using MT-based dosing outside the motor cortex.

Box 1. Common assumptions of TMS dosing based on motor cortex excitability for non-motor areas.

MT- and e-field-based TMS dosing individualize the stimulation strength based on motor-cortex excitability. When targeting non-motor regions, such as higher association cortices, with any of these dosing strategies, several—usually implicit—assumptions are made about the mechanisms that underlie stimulation effects.

1. **Skin-cortex distance**: MT-based dosing assumes similar skin-cortex distances for the motor hotspot in the primary motor cortex (M1) and the stimulation target. Only for similar skin-cortex distances, cortical stimulation exposures are comparable across targets.
2. **Cortical stimulation thresholds:**All dosing strategies assume similar stimulation thresholds across the cortex, that is, the neuronal tissue at M1 and the stimulation target are assumed to have the same activation functions towards TMS pulses.
3. **Stimulation protocol:**Numerous studies use single-pulse TMS to quantify the motor cortex excitability but apply repetitive TMS (rTMS) to non-motor target areas, for example, to modulate cognitive functions. Currently, all dosing strategies assume one global TMS threshold, independent of the temporal dynamics of the stimulation pattern (e.g., single-pulse TMS vs. rTMS).
4. **Outcome measure:**Many studies use similar cortical stimulation intensities (e.g., 100% of the resting motor threshold) to define motor cortex excitability (quantified via motor evoked potentials) and modulate behavioral responses (usually quantified as changes in response speed or accuracy) in cognitive experiments. Here, the same cortical stimulation threshold is assumed across different functional domains and outcome metrics.

Similar rationales apply when using dosing strategies based on other cortical excitability estimates, such as the phosphene threshold in the visual cortex.

## 2 Advantages of E-Field-Based Dosing over Motor-Threshold-Based Dosing

The motor threshold (MT) concept, which originated in the early days of TMS, was intended to standardize TMS effects across individuals and prevent adverse effects from overstimulation by individualizing the stimulation strength (Wassermann, 1998). The MT quantifies the*stimulator*device output (in % MSO) to yield muscle twitches (motor evoked potentials; MEPs) when stimulating the primary motor region (M1) with single TMS pulses. Despite its motor-centric definition, MT-based dosing is also commonly used for targets outside the motor cortex (Turi et al., 2021). A rationale for this generalization is the lack of a direct output measure to quantify the excitability of most non-motor regions. Critically, however, the*stimulator*output can only provide a rough estimate of the cortical*stimulation*exposure, which drives the cortical stimulation effects and is determined by the e-field magnitude and orientation at the cortex (Numssen et al., 2021). This poses various problems for MT-based dosing, most prominently for targets with a different skin-cortex distance than M1 (Koponen & Peterchev, 2020) or other macroscopic differences, such as different cortical thickness, yielding different cortical stimulation exposures for the same stimulator intensity. Likewise, the motor threshold does not correlate with the phosphene threshold (a metric for visual cortex excitability), when quantified via the*stimulator*intensity (Boroojerdi et al., 2002;Stewart et al., 2001). This illustrates that stimulation intensities for non-motor areas based on the motor threshold are somewhat arbitrary. E-field-based dosing approaches aim to solve this issue by normalizing the cortical*stimulation*strength across cortical areas. This can be achieved by computing the induced e-fields for both M1 and the actual target, and then determining the*stimulator*intensity required to produce the same cortical*stimulation*strength at the target area as in M1 at the motor threshold (seeCaulfield et al., 2021afor a similar e-field dosing implementation):

$$\begin{array}{l} \mathrm{Intensity}\;\mathrm{scaling}\;\mathrm{factor}\;=\;\mathrm{e-field}\;\mathrm{at}\;\mathrm{motor}\;\mathrm{hotspot}\;(\;\;\mathrm{V/m})\\ \;\;\;\;\;\;\;\;\;\;\;\mathrm{/e-field}\;\mathrm{at}\;\mathrm{target}\;(\;\;\mathrm{V/m}) \end{array}$$

$$\begin{array}{l} \mathrm{Stimulator}\;\mathrm{intensit}y\;(\%\;MSO)=\;\;MT\;(\%\;MSO)\\ \;\;\;\;\;\;\;\;\;\;\;\;\;\;\;*\;\mathrm{intensity}\;\mathrm{scaling}\;\mathrm{factor} \end{array}$$

That is, the e-field strength in M1 at MT is used as a proxy for the individual*cortical stimulation threshold*—the cortical stimulation strength required to modulate neuronal processing with TMS.

To evaluate the effectiveness and feasibility of e-field-based dosing across various cortical targets, we utilized this approach in 18 healthy participants (10 female; mean age = 30.5 years, SD = 5.88) to target different sensory-motor regions as well as higher-level association areas in the left hemisphere (Kuhnke, Numssen, et al., 2023). Sensory-motor regions included M1, somatomotor, and auditory cortices, while higher-level association regions included the inferior parietal lobe (IPL) and dorsolateral prefrontal cortex (DLPFC), which are common targets in TMS studies of cognition and for clinical applications (e.g.,Beynel et al., 2019;Cash et al., 2021). This approach was ideal to elucidate potential advantages and drawbacks of e-field-based dosing in comparison to MT-based dosing (e.g.,Kuhnke et al., 2020) and other dosing strategies (Stokes et al., 2005).

Standard MT-based dosing applies the same*stimulator*intensity (in % MSO) at all (motor and non-motor) targets. Here, we used 100% resting MT intensity to allow for straightforward comparisons between targets, instead of applying a fraction of MT as is frequently done (e.g., 90% or 110% MT;Beynel et al, 2019;Kuhnke et al., 2017). As a second approach, we used a method proposed byStokes et al. (2005)which aims to correct for cortical depth differences of motor- and non-motor-targets without assessing on the induced e-field. In Stokes’ approach, MT is adjusted by 3% MSO for every millimeter difference in skin-cortex distance between M1 and the target.

Like MT-based dosing and the Stokes approach, e-field-based dosing depends on the correct localization of the hand muscle representation to accurately extract the cortical stimulation threshold. To this end, we employed a state-of-the-art TMS motor mapping protocol (Weise, Numssen et al., 2020;Weise, Numssen, et al., 2022) to precisely locate the participant-specific first dorsal interosseous muscle representation in the primary motor cortex (M1) (seeSupplementary Materialsfor details). Subsequently, we computed the optimal coil position for the motor hotspot and experimentally measured the resting MT with this coil position (i.e., the stimulator intensity required to elicit 5 out of 10 MEPs of size ≥50 µV). This allowed us to precisely quantify the cortical stimulation threshold (defined as resting MT) in terms of the cortical electric field strength in V/m at the motor hotspot instead of the hardware-dependent (Wang et al., 2023) stimulator intensity %MSO. Subsequently, we computed optimal coil positions for each of the four other targets (auditory, somatomotor, IPL, DLPFC). These non-motor targets were defined based on functional MRI localizers and meta-analyses, following standard procedures in current TMS studies on cognition. Please see*Remaining challenges*—*Target definitions*for potential future pathways to improve target selection and target delineation*.*Finally, we calculated the stimulator intensities required to elicit the same e-field strength in each target as in M1 at resting MT—the cortical stimulation threshold.

## 3 Dosing Comparison: Individual Level

**Fig. 2.**
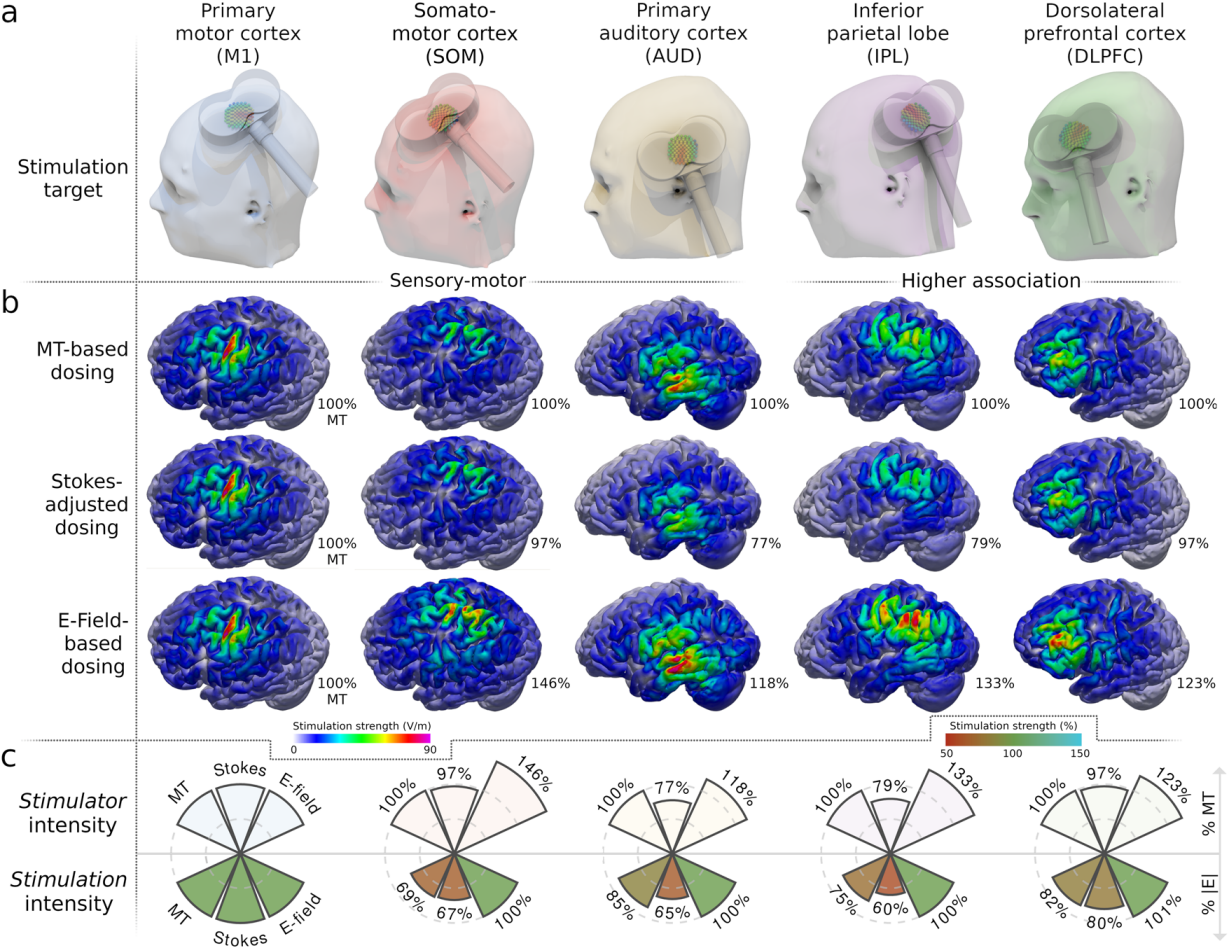
E-field dosing outperforms other dosing strategies on the participant level. (a) All coil placements were selected to maximize the cortical target stimulation. (b) Dosing based on the motor threshold (MT) alone (upper row) applies the same*stimulator*intensity across different cortical target regions (columns), yielding highly variable cortical*stimulation*strengths (quantified in volts per meter; V/m). The “Stokes” method (middle row) linearly adjusts the*stimulator*intensity for coil-to-target distance, but still results in a suboptimal match of cortical*stimulation*across targets. E-field-based dosing (bottom row) yields the same cortical*stimulation*strength for all targets. Color: |E|. Percentages: % of MT stimulator intensity. All e-fields are visualized on the gray matter surface for one representative participant. (c) The relationship between*stimulator*intensity (upper row) and cortical*stimulation*exposure (bottom row) differs strongly across cortical targets. The*stimulation*exposures were extracted at the cortical targets and related to M1 exposure at MT intensity (‘100%’).

Figure 2shows the comparison of the three dosing strategies for all targets in a representative individual participant. For M1 stimulation, the three dosing strategies do not differ as this stimulation target provides the common ground for the MT-based and e-field-based approaches (Fig. 2b, c, left column, Key Figure). M1 is effectively stimulated at 100% MT, leading to a hotspot of high cortical stimulation strength in the precentral gyrus. With MT-based dosing, the same stimulator intensity is simply applied to the other targets (e.g., 100% MT = 39% MSO for our example participant). While this approach seems to work reasonably well for the auditory cortex in this individual, the somatomotor cortex, IPL, and DLPFC seem to be understimulated relative to M1 (Fig. 2b, top row).

The Stokes approach yields lower stimulator intensities in our example participant for the non-motor targets (e.g., auditory: 77% MT; IPL: 79% MT) as these were located closer to the scalp than M1 (Fig. 2b, middle row). Crucially, however, the Stokes approach also leads to understimulation of the non-motor areas in this participant (Fig. 2b, middle row). The cortical stimulation exposure does not seem to be better matched between the non-motor targets and M1 than for MT-based dosing. Importantly, e-field-based dosing leads to substantially different*stimulator*intensities for the different targets in our example participant (Fig. 2b, bottom row). For instance, the somatomotor cortex needs to be stimulated at 146% MT (i.e., 57% MSO) to reach the cortical stimulation threshold for this participant.

Finally, e-field-based dosing precisely matches the effective cortical stimulation for the different targets (Fig. 2b, bottom row). For each target, e-field-based dosing yields a hotspot of high cortical stimulation strength. Comparisons of the*stimulator*intensity and the cortical*stimulation*exposure identify strong differences between cortical regions (Fig. 2c).

In conclusion, MT- and Stokes-based dosing fail to match the cortical stimulation intensity between different cortical targets within the same individual. In contrast, e-field dosing matches the cortical stimulation intensities for all the different targets (somatomotor, auditory, IPL, DLPFC) within our example participant to their individual cortical stimulation threshold (i.e., the e-field strength in M1 at rMT). Only e-field-based dosing allows to equalize the stimulation exposure across different cortical areas within the same individual.

## 4 Dosing Comparison: Group Level

**Fig. 3.**
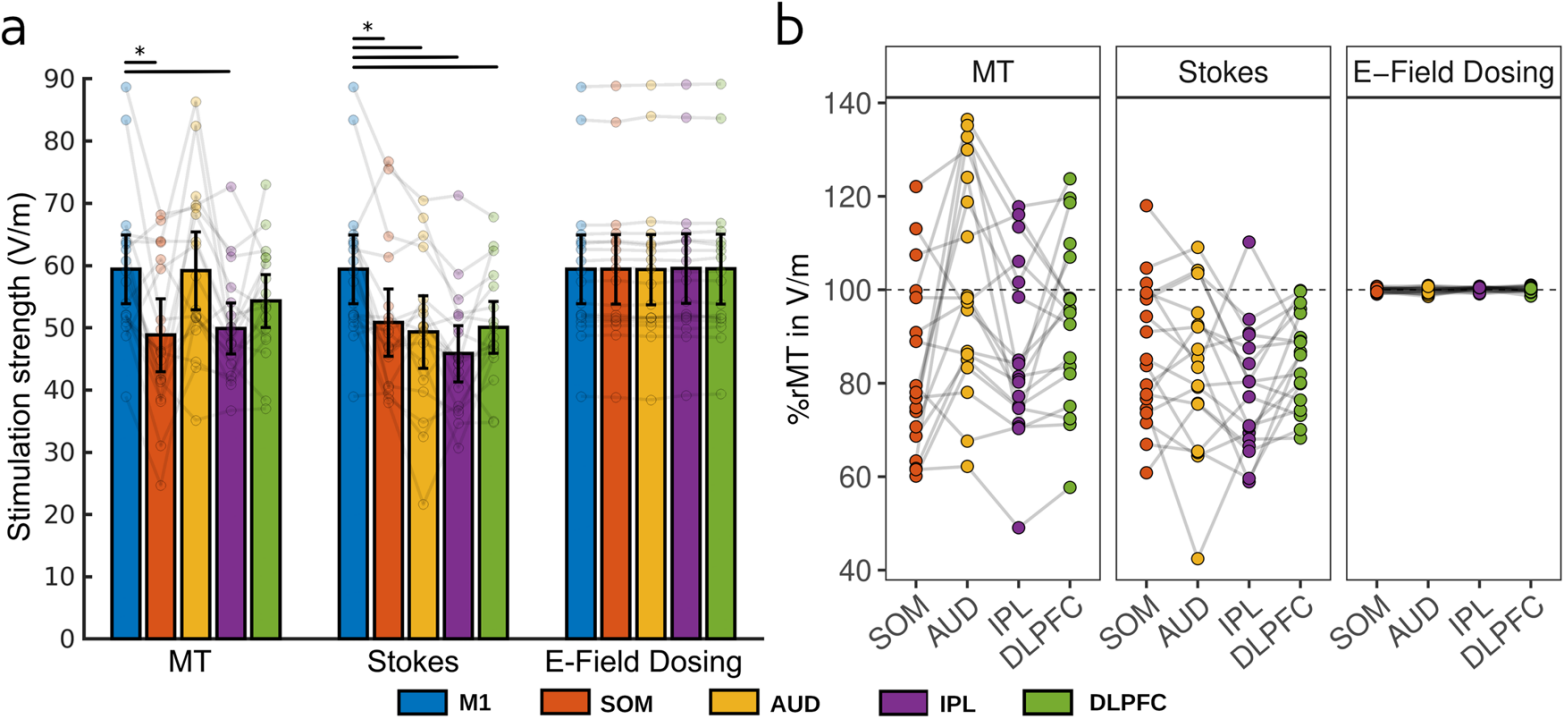
E-field-based dosing outperforms other dosing strategies on the group level. (a) The cortical stimulation strength (in V/m) is highly variable for MT-based dosing (left) and Stokes-adjusted dosing (middle), both within and between the 18 participants across the different cortical targets. In contrast, e-field-based dosing (right) yields the same stimulation strength for all targets within each participant, which also minimizes the between-participant variability. The cortical stimulation threshold centers around 60 V/m. (b) MT-based dosing fails to replicate cortical stimulation thresholds (y-axis) at other cortical targets, and Stokes-adjusted dosing only slightly improves this relationship. E-field-based dosing matches the cortical stimulation exposure at all cortical targets to the cortical stimulation threshold measured at M1. E-field magnitudes (|E|) were extracted and averaged within spherical ROIs (r = 5 mm) from gray matter volume only. Connected dots show individual participant data; error bars represent the 95% confidence interval. Black bars indicate significant differences (p < 0.05; Bonferroni-Holm corrected for multiple comparisons).

Results on the group level (obtained from n = 18 healthy volunteers) parallel the central findings from the representative single-person case inFigure 2. MT-based and Stokes-adjusted dosing lead to strong variability of cortical stimulation strengths, both within individuals across targets and between individuals for the same target (Fig. 3a). In contrast, e-field-based dosing matches the cortical stimulation strength for each target to the respective participant’s cortical stimulation threshold—the individual stimulation strength in M1 at MT (Fig. 3b)—minimizing within-participant variability. Minor deviations of the obtained e-field between targets within the same participant are caused by limitations of the stimulator device, whose intensity can only be changed in steps of 1% MSO. E-field dosing not only minimizes the within-participant variability but also reduces the between-participant variability, so that each cortical target receives the same stimulation strength on average (Fig. 3aright).

The average cortical stimulation threshold across participants was 59.5 V/m (SE = 2.8). For 15 out of 18 participants, the identified cortical threshold lies between 50 and 70 V/m, with one participant below and two participants above this range. Interestingly, cortical stimulation thresholds and model fits (between e-field and MEP magnitudes) are negatively correlated (r = -0.564, p = 0.019; seeFig. S1). That is, participants with a better model fit have lower estimated cortical thresholds. Distinct differences in model fits might point to inaccuracies in the modeling pipeline or suboptimal data sampling. With several parameters of the modeling pipeline currently being subject to refinement, such as improved tissue segmentations, better group-based and individual estimates of tissue conductivities, and the impact of gray matter density on submillimeter e-field warps, the overall simulation accuracy will potentially be further improved in the future (see*Remaining challenges of e-field-based dosing*—*Simulation accuracy*section).

In conclusion, e-field based dosing better matches the cortical stimulation strength across the cortex than MT-based dosing approaches—both within and across individuals. Whereas e-field dosing matches the induced e-fields by design, MT and Stokes-based dosing fail to match the induced e-fields between different targets and individuals. Therefore, e-field-based dosing may increase the stimulation efficacy and reduce both the within- and between-person variability of TMS effects. Overall,*a priori*e-field simulations promise to substantially improve TMS studies with non-motor target areas.

## 5 Remaining Challenges of E-Field-Based Dosing

**Fig. 4.**
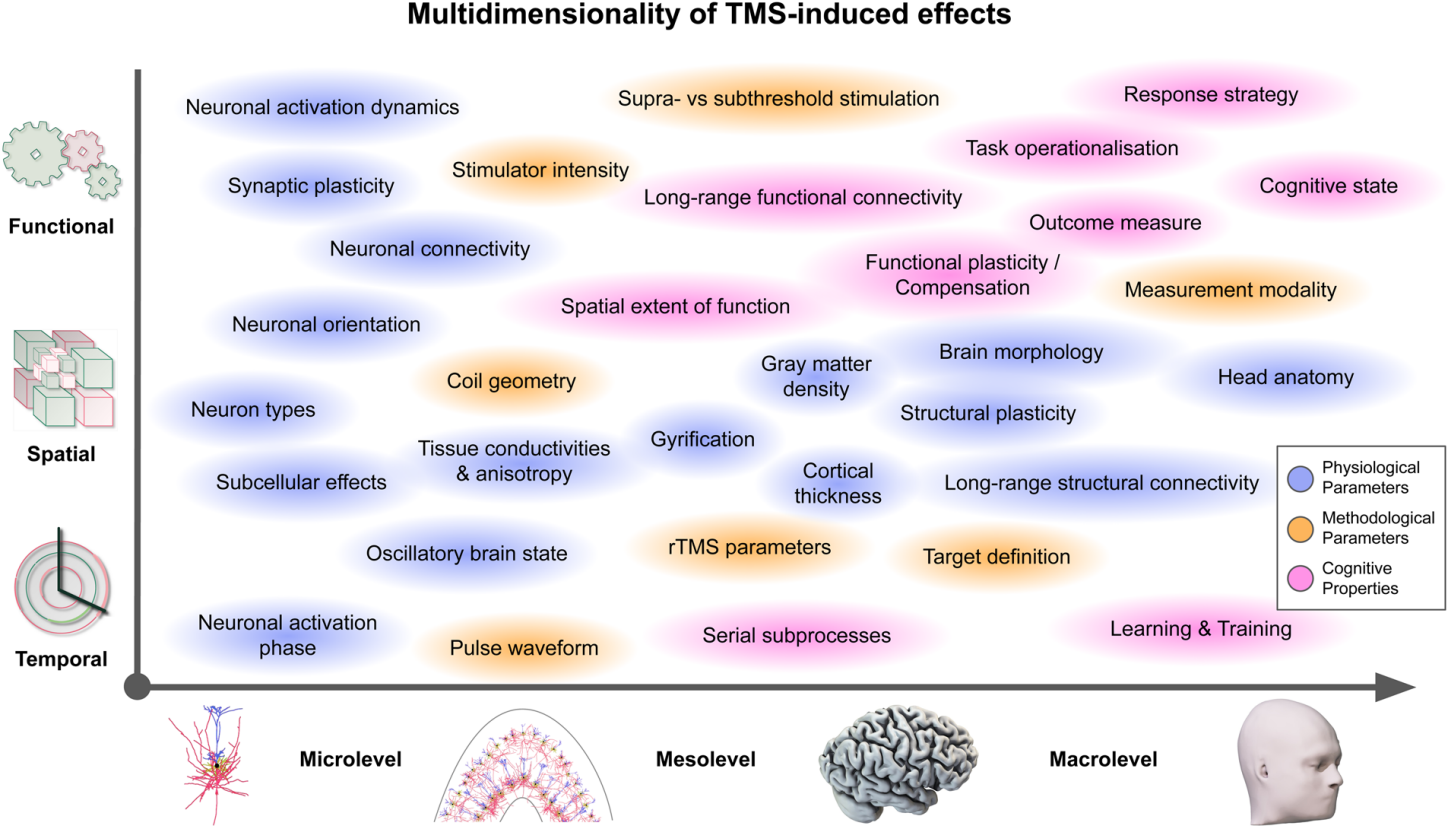
TMS-induced effects depend on a multidimensional set of factors. The outcome of a TMS study (or therapeutical intervention) is the sum of various factors, spanning**physiological parameters**(blue) of the individual (such as gyrification patterns and electric tissue properties),**methodological parameters**(orange; such as pulse waveform and target definition), and**cognitive properties**(magenta; such as response strategies and cognitive brain state). These are defined in the**temporal domain**(*microlevel*: different neuronal activation phases might yield different neuronal responses to a TMS pulse;*mesolevel*: stimulating during different serial subprocesses of a function;*macrolevel*: different levels of training render different cortical target regions effectively), the**spatial domain**(*microlevel*: neuronal orientation towards the induced e-field;*mesolevel*: the TMS coil geometry and gyrification patterns define the induced e-field;*macrolevel*: long-range white matter fiber tracts allow for distal TMS effects), or the**functional domain**(*microlev*el: single-cell activation mechanisms;*mesolevel*: supra- vs. subthreshold stimulation;*macrolevel*: outcome measure, such as response speed vs. accuracy).

Despite significant improvements of e-field-based dosing from conceptual and methodological perspectives, several challenges in the complex interplay between physiological, methodological, and cognitive parameters (Fig. 4) remain to be addressed (Box 2). This includes assessment to test the behavioral and clinical relevance in experimental and clinical studies. Importantly, these crucial parameters, as detailed below, should not be considered in isolation but should instead be seen as a set of factors that together define the TMS-induced effects and may interact with each other.

Box 2. Outstanding Questions.

- Does the cortical threshold for effective stimulation differ between primary regions and higher-level association areas? How large is the impact of cytoarchitectonic differences between regions on a stimulation threshold?
- Do cortical stimulation thresholds differ across individuals? Are thresholds stable within an individual across the lifespan? What are the physiological factors influencing these thresholds?
- Can a cortical stimulation threshold measured with single-pulse TMS be transferred to repetitive TMS protocols for the study of cognition?
- How does the cortical stimulation threshold interact with the current brain state?

### 5.1 Simulation accuracy

The development of easy-to-use toolboxes to compute the induced e-fields for individual anatomies and coil placements is still in its infancy and the field has not yet settled on common grounds. For example, the number of relevant tissue types that need to be assessed during e-field computation is still debated (Weise, Wartman, et al., 2022). In addition, although considerable variations of relevant physiological properties such as electric conductivities across individuals and across the lifespan have been reported (Antonakakis et al., 2020;Wagner et al., 2004), these variations are currently not included in field modeling toolboxes. Approaches to estimate person-specific tissue conductivities*in vivo*are under development (e.g., magnetic resonance current density imaging; MRCDI; magnetic resonance electrical impedance tomography; MREIT;Eroğlu et al., 2021;Göksu et al., 2018;Yazdanian et al., 2020). Importantly, these variance sources do not necessarily impede within-participant comparisons and e-field dosing based on individualized M1 stimulation thresholds as these inaccuracies are constant within individuals and toolboxes. Instead, these limitations affect across-toolbox comparisons and considerations about mesoscopic stimulation mechanistics, which rely on physically correct e-field computations, including precise information about the pulse shape. Due to these inaccuracies, the generalizability of individual cortical field thresholds, such as the ~60 V/m estimate presented above, remains to be tested.

### 5.2 Target definitions

E-field-based dosing crucially depends on the correct definition of the cortical M1 region-of-interest (ROI) since the effective e-field that yields a neuronal effect is measured at this location. Specifically, the ROI location, parameters (ROI shape, size, etc.), and summary statistics (e.g., 99% percentile, maximum, etc.) influence this quantity and the brain stimulation community has yet to settle on common values for these parameters (seeVan Hoornweder et al., 2023for an overview on the different ROI definitions and extraction approaches used in TMS studies). Likewise, the “real” cortical target needs to be defined accurately, again, both with respect to its participant-specific location and spatial extent. However, in TMS studies of cognition, the cortical targets are often defined based on group-level fMRI or even meta-analyses (Beynel et al., 2019), ignoring individual differences, and thus, yielding suboptimal e-field calibrations. Besides participant-specific functional MRI localizers for TMS studies on cognition (Sack et al., 2009), promising strategies have been proposed that leverage individual functional connectivity and resting-state network mappings to define cortical targets (Balderston et al., 2022;Lueckel et al., 2023;Lynch et al., 2022). These approaches might also open the door towards defining off-targets, that is, regions that should explicitly not be stimulated. Aside from a spatial target definition, the temporal target (Fig. 4, bottom;Romei et al., 2016) also needs to be defined accurately to effectively modulate cortical processing of a specific function, potentially ranging from milliseconds (oscillatory states;Bergmann et al., 2012;Siebner et al., 2022) to seconds (sequential subprocesses of a function;Sack et al., 2005) to even longer time periods (adaptations due to learning and long-term plasticity or compensation;Bergmann & Hartwigsen, 2021).

### 5.3 Different TMS protocols

Besides issues regarding the spatial distribution of the induced e-field, it is worth noting that while the motor threshold is typically assessed using single-pulse TMS, repetitive TMS (rTMS) is commonly employed in non-motor studies. For example, TMS for major depression therapy utilizes various different protocols, including 1 Hz to 20 Hz rTMS and several variations of theta-burst stimulation (Chen et al., 2013;Teng et al., 2017;Voigt et al., 2021). However, the mesoscopic effects of different rTMS protocols and, thus, their relation to MT thresholds based on single-pulse TMS are currently unknown. To gain a deeper understanding of this issue, modeling approaches will potentially provide valuable insights (see, e.g., NeMo-TMS toolbox;Shirinpour et al., 2021). Currently,*cortical stimulation thresholds*are derived from a variety of experimental and modeling approaches, resulting in high variability. For example, experimental approaches identified thresholds from 35 V/m to 60 V/m when quantified via EEG (double pulses,Rosanova et al., 2009; individual alpha frequency,Zmeykina et al., 2020) and from 60 to above 100 V/m when measured with EMG (single pulses;Kuhnke, Numssen, et al., 2023;Numssen et al., 2021). In contrast, modeling approaches and in vitro experiments identified thresholds between 120 V/m and 300 V/m (Aberra et al., 2020,^2023^;Shirinpour et al., 2021;Turi et al., 2022;Weise et al., 2023). It is important to note that modeling approaches currently use simplified setups, such as isolated neurons instead of interconnected neuronal networks and other cell types, and that neuronal models originate from rodent samples (Weise et al., 2023). Different pulse waveforms, such as mono- vs. biphasic (Wendt et al., 2023) and steeper vs. longer pulses (Sommer & Paulus, 2023), add to the different neuronal activation dynamics (Koponen et al., 2018;Peterchev et al., 2013;Zeng et al., 2022). Further research in this area is needed to elucidate the relationships between cortical thresholds for single-pulse TMS and rTMS, particularly in non-motor regions.

### 5.4 Different cortical targets

It is important to note that—besides the implicitly assumed similarity between single-pulse TMS and rTMS thresholds (see above)—also implicit assumptions of similar cortical responses for different cortical regions are made in (non-motor) TMS studies. For example, the cortical tissue of M1 and IPL differs substantially on the mesoscopic level, with M1 having large amounts of giant Betz cells in layer V and no layer IV, and the IPL showing the opposite pattern (Caspers et al., 2013). Despite this variability of physiological properties, cortical thresholds are currently assumed to be similar across different cortical regions. Most critically, recent modeling approaches have shown that different physiological properties (e.g.,Dubbioso et al., 2021) and neuronal types, based on their morphology and orientation within the cortical tissue, show different coupling behavior to the induced electric field from TMS (Aberra et al., 2018,^2020^;Weise et al., 2023). It should be noted that also macroscopic differences affect the induced e-field, including differences in cortical thickness that require careful e-field quantification settings (e.g., surface vs. volume extraction). This questions the assumptions of similarity between cortical regions with respect to transcranial stimulation.

### 5.5 Different functional domains

Finally, in TMS studies of cognition, the existence of a single cortical excitation threshold across different functional domains is assumed implicitly. The motor threshold measures the minimum cortical excitation (with single-pulse TMS of the primary motor region) necessary to elicit MEPs just above the EMG noise floor. However, it remains unknown if the same cortical excitation threshold can be applied to effectively modulate other (cognitive) functions, such as attentional reorienting (Jing et al., 2023), sentence processing (Kuhnke et al., 2017;Meyer et al., 2018;van der Burght et al., 2023), or conceptual-semantic processing (Kuhnke et al., 2020,^2023^). As pre-activation of the motor cortex drastically lowers the cortical threshold to evoke MEPs (Rossi et al., 2009; seeHolmes et al., 2023for unintended side-effects), different (cognitive) brain states are also likely to affect stimulation effects within higher association cortices (Feurra et al., 2013;Krause et al., 2022;Silvanto et al., 2008seeHartwigsen & Silvanto, 2022for discussion). So far, relationships between functional domains and cortical excitation thresholds remain unknown.

## 6 Concluding Remarks

Although e-field-based dosing is no magic bullet for all challenges associated with NIBS studies, it has the potential to substantially decrease across- and within-person variance of cortical modulation in non-motor studies. By providing a more biologically plausible dosing metric, this approach can potentially play a crucial role in improving the personalization of TMS and tES treatments in clinical settings, as well as increasing the effect sizes of NIBS studies at the group level. Crucially, these assumptions have yet to be tested in experimental and clinical studies. As such, e-field-based dosing represents a promising avenue for future research in the field of NIBS.

## Supplementary Material

Supplementary Material

## Data Availability

Electric field values from the regions of interest and analysis scripts are publicly available:https://gitlab.gwdg.de/tms-localization/papers/dosing_strategies
