# Supplementary material for: Electric-field-based dosing for TMS
Source: Imaging Neurosci (Camb). 2024 Mar 11;2:imag-2-00106. doi: 10.1162/imag_a_00106 (PMC12247572; doi:10.1162/imag_a_00106)
Supplement: Supplementary Material [file imag_a_00106-supp.pdf]

Supplementary Material

Electric field based dosing for TMS

Ole Numssen[1,2]*, Philipp Kuhnke[1,3]*, Konstantin Weise[2,4], Gesa Hartwigsen[1,3]

[1] Lise Meitner Research Group Cognition and Plasticity, Max Planck Institute for Human Cognitive and Brain Sciences, Leipzig, Germany
[2] Methods and Development Group Brain Networks, Max Planck Institute for Human Cognitive and Brain Sciences, Leipzig, Germany
[3] Wilhelm Wundt Institute for Psychology, Leipzig University, Germany
[4] Department of Clinical Medicine, Aarhus University, Aarhus, Denmark

* shared first authors

Locating the motor hotspot with TMS

Like MT-based dosing and the Stokes approach, e-field based dosing depends on the correct localization of the hand muscle representation to accurately extract the cortical stimulation threshold. To this end, we employed a state-of-the-art TMS motor mapping protocol (Weise & Numssen et al., 2022) to precisely localize the participant-specific muscle representation in the primary motor cortex (M1). This protocol utilizes pyNIBS v0.3 (Numssen et al., 2021) and SimNIBS v4.0 (Saturnino et al., 2019; Thielscher et al., 2015): First, we constructed high-resolution head models based on individual T1-, T2-, and diffusion-weighted MR images (for details, see *Electric field modeling details* below). Second, we applied ~300 TMS pulses in a 2-3 cm radius around the presumptive motor hotpot, and recorded the coil positions/orientations and motor evoked potentials (MEPs) from the first dorsal interosseous muscle. Third, we simulated the TMS-induced e-field for each pulse using SimNIBS. Fourth, for each cortical element (in a region-of-interest of M1, premotor, and somatosensory cortices), we fit the local e-field magnitude onto the MEP magnitude and quantified the goodness-of-fit (R^2). Fifth, we identified the motor hotspot as the cortical element with the best fit (highest R^2) on the M1 gyral crown. Sixth, we computed the optimal coil position for the motor hotspot. To this end, we selected the coil position from a grid of 2-cm radius and 2-mm steps that maximized the e-field magnitude in a 5-mm sphere around the motor hotspot, restricted to gray matter (‘position optimization’; see Numssen et al., 2021 for details). Finally, we experimentally measured the resting MT at this optimal coil position (i.e., the stimulator intensity required to elicit 5 out of 10 MEPs of size ≥ 50 μV). This allowed us to precisely quantify the cortical stimulation threshold (defined as resting MT) in V/m at the cortical motor hotspot.

Electric field modeling details

For the electric field simulations with the finite element method (FEM, SimNIBS v4.0, Saturnino et al., 2019) we created participant-specific high-resolution head models based on individual T1-, T2-, and diffusion-weighted MR images (using the CHARM method; Puonti et al., 2020).

MR images were acquired using a 3T MRI scanner (Siemens, Erlangen, Germany) using the following sequences:

- **T1-weighted MPRAGE**: 176 slices in sagittal orientation; repetition time (TR): 2.3 s; echo time (TE): 2.98 ms; field of view: 256 mm; voxel size: 1 x 1 x 1 mm; no slice gap; flip angle: 9°; phase encoding direction: A/P
- **T2-weighted**: 192 slices in sagittal orientation; voxel size = 0.9 x 0.45 x 0.45 mm; flip angle = 120°; TR = 3.2 s; TE = 408 ms
- **diffusion-weighted**: 67 slices in axial orientation; matrix size = 128 x 128; voxel size = 1.7 x 1.7 x 1.7 mm; flip angle = 90°; TR = 7 s; TE = 80 ms, 67 diffusion directions, b-value 1000 s/mm3

T1- and T2-weighted images were used for segmenting the main tissues of the head: scalp, skull, grey matter (GM), white matter (WM), and cerebrospinal fluid (CSF). Diffusion-weighted images were employed to include anisotropic conductivity information. The electrical field was calculated for 1 A/μs, using default tissue conductivities values (σ_{scalp} = 0.465 S/m, σ_{skull} = 0.01 S/m, σ_{GM} = 0.275 S/m, σ_{WM} = 0.126 S/m, σ_{CSF} = 1.654 S/m; Thielscher et al., 2011; Wagner et al., 2004).

Sample Information

Table S1. Sample statistics - Individual participants

ID	Sex	Age	LQ	rMT	SOM Ratio	AUD Ratio	IPL Ratio	DLPFC Ratio	SOM %	AUD %	IPL %	DLPFC %
01	M	37	100	40	1.350	0.730	0.850	0.840	54	29	34	33
02	M	40	100	42	1.630	1.610	2.040	1.730	68	68	86	73
03	M	38	75	49	1.280	0.840	1.020	1.080	63	41	50	53
04	F	33	80	35	1.580	0.750	0.880	1.020	55	26	31	36
05	F	37	100	49	0.880	1.040	1.180	1.040	43	51	58	51
06	F	30	100	56	1.000	0.740	1.230	1.200	56	41	69	67
07	M	31	100	39	1.100	1.030	0.940	1.050	43	40	37	41
08	M	38	100	45	1.620	1.150	1.190	0.930	73	52	53	42
09	F	27	100	39	1.460	1.180	1.330	1.220	57	46	52	48
10	F	24	80	40	1.660	1.200	1.340	1.380	67	48	54	55
11	M	34	73	47	1.410	1.010	1.240	1.020	67	48	58	48
12	M	31	100	59	1.340	0.810	1.400	0.910	79	48	83	54
13	F	25	80	50	1.020	1.020	1.300	1.170	51	51	65	59
14	F	25	87	57	0.930	0.900	0.860	1.020	53	51	49	58
15	M	26	100	56	0.820	0.770	0.980	0.810	46	43	55	45
16	F	22	75	56	1.300	1.280	1.420	1.330	73	72	80	75
17	F	22	100	44	1.120	1.160	1.250	0.840	49	51	55	37
18	F	29	85	32	1.260	1.480	1.420	1.400	40	47	45	45

Notes: LQ: Handedness laterality quotient. rMT = resting motor threshold in % MSO (maximum stimulator output). SOM = somatomotor cortex; AUD = auditory cortex; IPL = inferior parietal lobe; DLPFC = dorsolateral prefrontal cortex. SOM Ratio, AUD Ratio, IPL Ratio, and DLPFC Ratio columns provide scaling factors for e-field based dosing with respect to the resting motor threshold (rMT). SOM %, AUD %, IPL %, DLPFC % columns provide the resulting stimulator intensities in % MSO.

Table S2. Sample statistics - Group level

	Age	LQ	rMT	SOM Ratio	AUD Ratio	IPL Ratio	DLPFC Ratio	SOM %	AUD %	IPL %	DLPFC %
**MEAN**	30.50	90.83	46.39	1.264	1.039	1.215	1.111	57.61	47.39	56.33	51.11
**SD**	5.88	11.01	8.15	0.268	0.255	0.285	0.237	11.58	11.00	15.66	12.06
**MIN**	22	73	32	0.820	0.730	0.850	0.810	40	26	31	33
**MAX**	40	100	59	1.660	1.610	2.040	1.730	79	72	86	75

Notes: LQ: Handedness laterality quotient. rMT = resting motor threshold in % MSO (maximum stimulator output). SOM = somatomotor cortex; AUD = auditory cortex; IPL = inferior parietal lobe; DLPFC = dorsolateral prefrontal cortex. SOM Ratio, AUD Ratio, IPL Ratio, and DLPFC Ratio columns provide scaling factors for e-field based dosing with respect to the resting motor threshold (rMT). SOM %, AUD %, IPL %, DLPFC % columns provide the resulting stimulator intensities in % MSO.

Relationship between cortical stimulation thresholds & modeling fits

In the main text, we argue that exceptionally high cortical stimulation thresholds in some participants (as compared to other participants) may be due to suboptimal modeling, rather than genuinely higher stimulation thresholds in these individuals. This notion is supported by a negative correlation between cortical stimulation thresholds (i.e., the e-field strength in the M1 hotspot at resting MT; V/m) and modeling fits between the e-field magnitude and MEP amplitude at the motor hotspot (R^2) (Figure S1). In other words, participants with worse modeling fits show higher cortical stimulation thresholds ($r = -0.56$, $p = 0.019$).

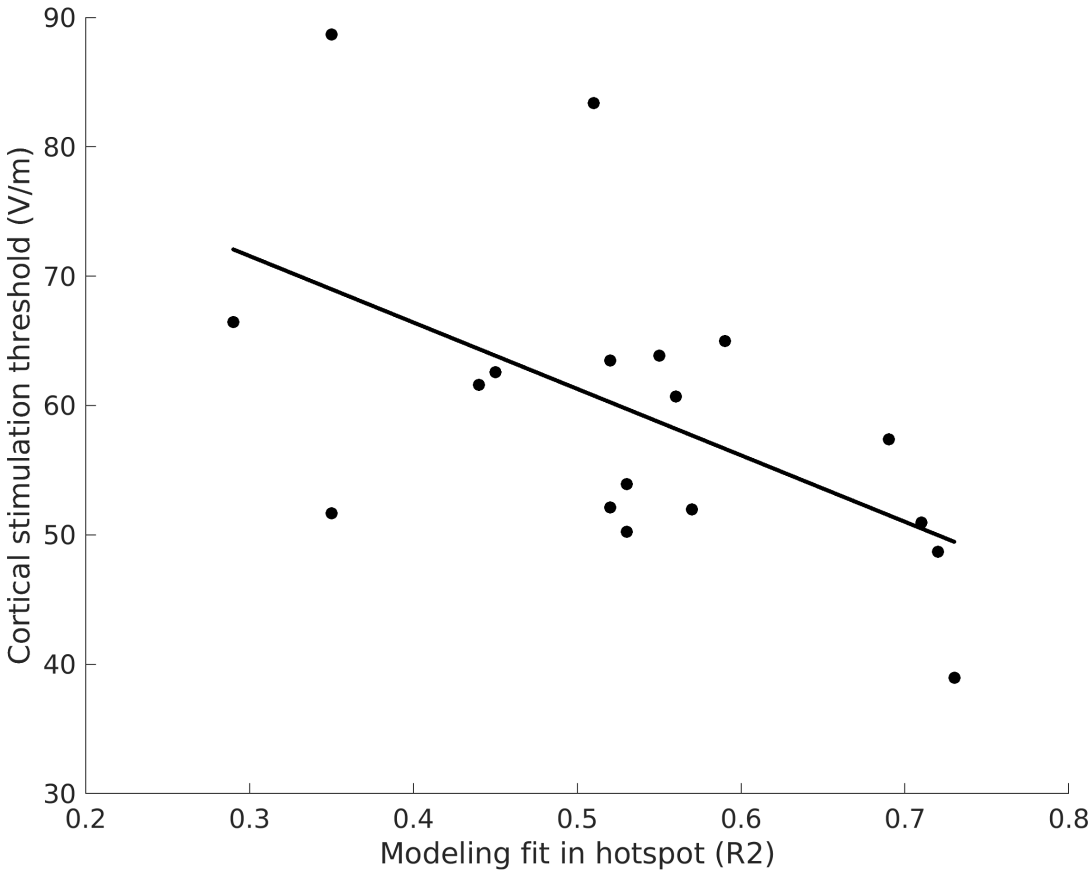

Figure S1. Correlation of individual fits between the e-field magnitude and MEP amplitude at the motor hotspot (R^2) and cortical stimulation thresholds (V/m).

References

Numssen, O., Zier, A. L., Thielscher, A., Hartwigsen, G., Knösche, T. R., & Weise, K. (2021). Efficient high-resolution TMS mapping of the human motor cortex by nonlinear regression. *NeuroImage*, *245*, 118654. DOI: 10.1016/j.neuroimage.2021.118654

Puonti, O., Van Leemput, K., Saturnino, G. B., Siebner, H. R., Madsen, K. H., & Thielscher, A. (2020). Accurate and robust whole-head segmentation from magnetic resonance images for individualized head modeling. *NeuroImage*, *219*, 117044. DOI: 10.1016/j.neuroimage.2020.117044

Saturnino, G. B., Puonti, O., Nielsen, J. D., Antonenko, D., Madsen, K. H., & Thielscher, A. (2019). SimNIBS 2.1: A Comprehensive Pipeline for Individualized Electric Field Modelling for Transcranial Brain Stimulation. In: Makarov, S., Horner, M., Noetscher, G. (Eds.), *Brain and Human Body Modeling* (3-25). Springer, Cham. DOI: 10.1007/978-3-030-21293-3_1

Thielscher, A., Antunes, A., & Saturnino, G. B. (2015). Field modeling for transcranial magnetic stimulation: A useful tool to understand the physiological effects of TMS? *Annu. Int. Conf. IEEE Eng. Med. Biol. Soc., 2015,* 222–225. DOI: 10.1109/embc.2015.7318340

Wagner, T. A., Zahn, M., Grodzinsky, A. J., & Pascual-Leone, A. (2004). Three-dimensional head model simulation of transcranial magnetic stimulation. *IEEE Trans. Biomed. Eng.*, *51*(9), 1586–1598. DOI: 10.1109/TBME.2004.827925

Weise, K., Numssen, O., Kalloch, B., Zier, A. L., Thielscher, A., Haueisen, J., Hartwigsen, G., & Knösche, T. R. (2022). Precise motor mapping with transcranial magnetic stimulation. *Nat. protoc.*, *18*(2), 293–318. DOI: 10.1038/s41596-022-00776-6
